# Establishment of a novel cell line from a rare human duodenal poorly differentiated neuroendocrine carcinoma

**DOI:** 10.18632/oncotarget.26367

**Published:** 2018-11-23

**Authors:** Kazuyoshi Yanagihara, Takanori Kubo, Keichiro Mihara, Takeshi Kuwata, Atsushi Ochiai, Toshio Seyama, Hiroshi Yokozaki

**Affiliations:** ^1^ Division of Biomarker Discovery, Exploratory Oncology and Clinical Trial Center, National Cancer Center, Chiba, Japan; ^2^ Department of Life Sciences, Yasuda Women's University Faculty of Pharmacy, Hiroshima, Japan; ^3^ Department of Hematology/Oncology, Research Institute for Radiation Biology and Medicine, Hiroshima University, Hiroshima, Japan; ^4^ Department of Pathology and Clinical Laboratories, National Cancer Center Hospital East, Chiba, Japan; ^5^ Division of Pathology, Department of Pathology, Kobe University Graduate School of Medicine, Kobe, Japan

**Keywords:** BRAF^V600E^ mutation, duodenal neuroendocrine carcinoma, established cell line, orthotopic animal model, rare human cancer

## Abstract

Poorly differentiated neuroendocrine carcinoma of the duodenum (D-NEC) is a rare cancer with poor prognosis. However, a D-NEC cell line has not yet been established to study the disease. We established a cell line, TCC-NECT-2, from the ascites tumor of a 59-year-old male Japanese patient with D-NEC. TCC-NECT-2 was positive for neuroendocrine markers, chromogranin A (CGA), cluster of differentiation 56 (CD56/NCAM), synaptophysin (SYN/p38), and neuron specific enolase (NSE). Cells exhibited retinoblastoma (RB) protein loss. Orthotopic implantation of TCC-NECT-2 cells into nu/nu mice resulted in tumor formation (incidence = 83.3%) with neuroendocrine characteristics, metastasis, and weight loss. BRAF^V600E^ and TP53 mutations and C-MYC gene amplification were also observed in TCC-NECT-2. BRAF^V600E^-expressing TCC-NECT-2 cells were sensitive to BRAF inhibitor vemurafenib, and especially dabrafenib, *in vitro*, and were strongly inhibited in a dose-dependent manner. Dabrafenib treatment (30 mg/kg) in a xenograft model for 14 days significantly suppressed tumor growth (percent tumor growth inhibition, TGI% = 48.04). An enhanced therapeutic effect (TGI% = 95.81) was observed on combined treatment of dabrafenib and irinotecan (40 mg/kg). Therefore, TCC-NECT-2, the first reported cell line derived from D-NEC, might serve as a useful model to study the basic biology of D-NEC and translational applications for treatment.

## INTRODUCTION

According to the revised WHO-classification from 2010, which is based on the mitotic count and Ki-67 index, neuroendocrine tumors (NETs) are classified as well-differentiated neuroendocrine grade 1 tumors (NET G1: < 2 mitoses/10 high-power fields; Ki-67 index ≤ 2%), moderately differentiated grade 2 tumors (NET G2: 2–20 mitoses/10 high-power fields; Ki-67 index 3–20%), poorly differentiated and clinically highly aggressive grade 3 large- or small-cell type neuroendocrine carcinoma (NEC G3: > 20 mitoses/10 high-power fields; Ki-67 index > 20%), mixed adeno-NEC, and hyperplastic and pre-neoplastic lesions [1, 2]. Whereas tumor differentiation was not emphasized in the previous 2010 classification scheme, the newly published WHO 2017 classification defines the well-differentiated subtype as neuroendocrine tumor grade 3 (NET G3), and separates it from poorly differentiated subtypes [3]. Poorly differentiated NEC is morphologically composed of small-cell, large-cell, and mixed types. Although the gastroenteropancreatic (GEP) tract is the most common site for NEC outside the lung [4, 5], a large-scale European database indicates that GEP-NEC accounts for only 8% of malignant digestive endocrine neoplasms [6].

Duodenal NETs (D-NETs) are rare tumors with an overall prevalence of 0.17/100,000 individuals in Japan [7, 8]. Based on the WHO-classification, D-NETs are classified as NET G1 (50–75%), NET G2 (25–50%), and NEC (≤ 3%). Similarly, the incidence of primary NEC of the duodenum (D-NEC) is very low. As an aggressive GEP carcinoma, D-NEC is a rapidly progressing disease that frequently metastasizes to regional lymph nodes and the liver, and is associated with a very poor prognosis [8]. To date, the only curative treatment option is early surgery with radical tumor resection. The current standard treatment for NEC comprises combinatorial chemotherapy with platinum-based drugs and etoposide; however, this treatment has demonstrated disappointing results [9, 10].

Preclinical xenograft models of established GEP carcinoma cell lines that show human-like tumor progression help to characterize the disease process and develop novel therapeutic approaches. Previously, our reports based on the orthotopic implantation (OI) of gastric carcinoma cells showed that subsequent tumor growth results in peritoneal dissemination and metastases to various organs, similar to that observed in human cases [11]. We also recently reported a metastatic mouse model of OI using human gastric carcinoma cells. This model effectively mimics the tumor/host interaction and pathogenesis [12].

Preclinical studies are essential to understand the cell biology of disease and discover new agents. Thus, the lack of adequate experimental models for human D-NEC [13] prompted us to generate new cell lines that could be used to study the pathogenesis of this disease. Here, we describe the establishment and characterization of a human D-NEC cell line, TCC-NECT-2. Moreover, we report the anti-tumor activity of a BRAF inhibitor, as well as that in combination with irinotecan, using our BRAF^V600E^-harboring TCC-NECT-2 xenograft model.

## RESULTS

### Establishment and characterization of the human D-NEC, TCC-NECT-2 cell line

**Figure 1:**
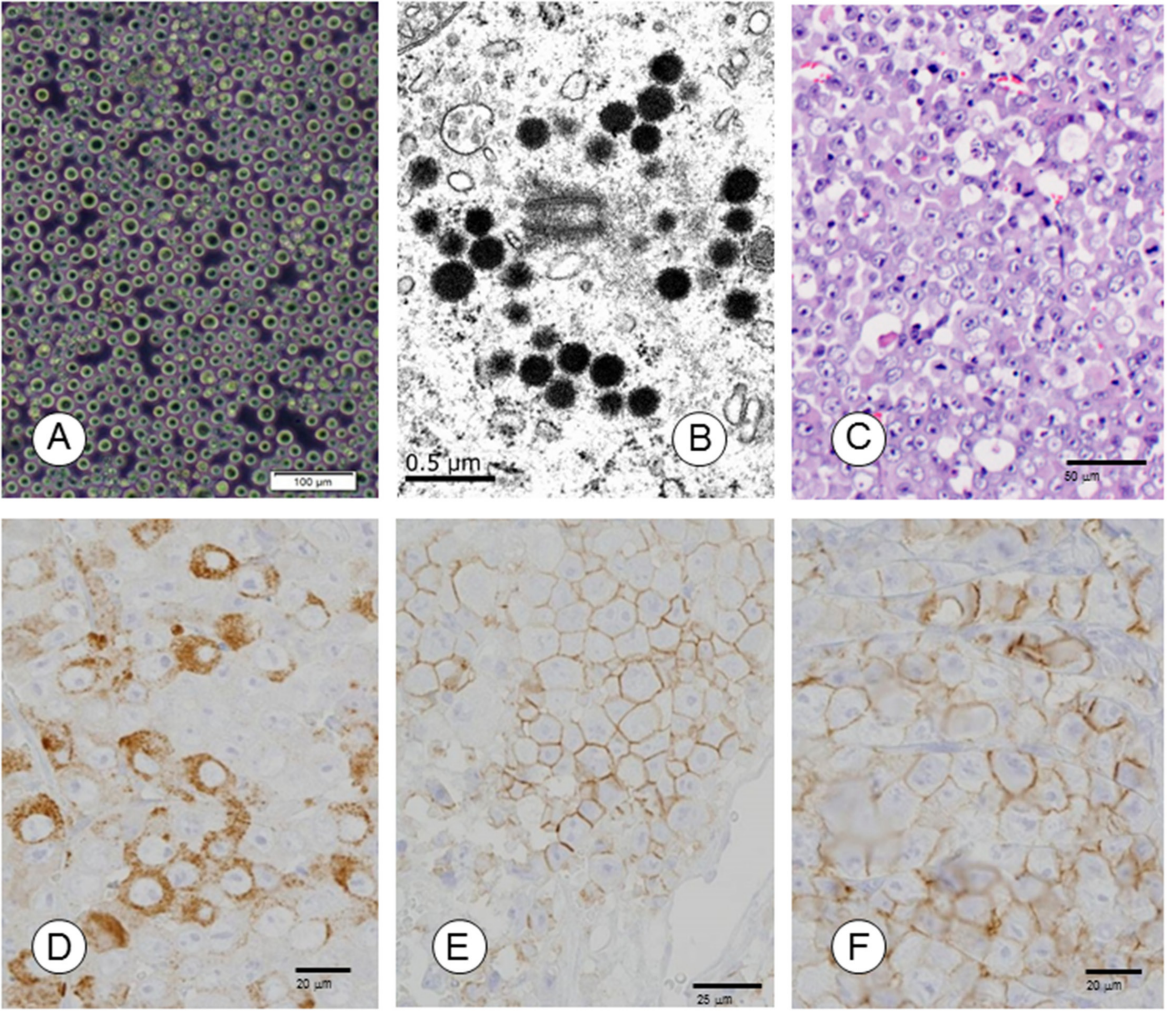
Morphological and immunohistochemical characterization of TCC-NECT-2 cell line **(A)** Phase-contrast photomicrographs of TCC-NECT-2 cells. Scale bar: 100 μm. **(B)** Electron-microscopy revealed electron-dense core neurosecretory granules in TCC-NECT-2 cells. Scale bar: 0.5 μm. **(C)** Photomicrographs of subcutaneous tumors in recipient nu/nu mice following subcutaneous (s.c.) injection of TCC-NECT-2 cells. Scale bar: 50 μm, hematoxylin–eosin (HE) staining. **(D)** CGA, Scale bar: 20 μm, **(E)** CD56/NCAM, Scale bar: 25 μm, and **(F)** SYN/p38, Scale bar: 20 μm were immunohistochemically evaluated as indicated.

The TCC-NECT-2 cell line derived from human NEC of the duodenum was newly established. TCC-NECT-2 cells had spherical cell morphology, floated freely, and showed a characteristic pattern of weak attachment (Figure 1A). The cell line was anchorage-independent (63.2% efficiency), and the doubling time was approximately 31.4 hours in RPMI1640 medium supplemented with 10% FBS. The Ki-67 index was 68.4% (Table 1).

**Table 1:** Biological characteristics of newly established neuroendocrine carcinoma of the duodenum (D-NEC) cell line TCC-NECT-2

Cell line	Origin		Growth^*^			Neuroendocrine tumor marker^#^						Tumor marker^+^		
	Age/sex	Tumor source	Pattern/DT(h)	In CDM/agar (%)	Ki-67 index (%)	NSE (ng/mL)	CGA	CD56/NCAM	SYN/p38	SSTR	RB	CEAng/mL	CA19-9U/mL	CA125 U/mL
TCC-NECT-2	59/M	Ascites	F/31.4	(-)/63.2	68.4	5.2	(+)	(+)	(+)	(-)	(-)	ud	41.6	ud

^*^F, floating type; DT, the doubling time of each line was determined as described previously (12); Chemical defined medium (CDM), composed of DMEM/Ham's F-12 (1:1) medium supplemented with 0.05% BSA; (+), positive; (-), negative; ud, undetectable. Colony formation on semisolid agar was assayed by plating 10^3^–10^4^ cells in DMEM containing 10% FBS and 0.33% Difco noble agar. The number of colonies formed was counted 21 days after cell plating (PE, %). The expression of Ki-67 was detected by immunohistochemical staining as described in the Materials and Methods section.

^#^Secretion of NSE was tested in culture fluids by chemiluminescent enzyme immunoassay (CLEIA) as described in the Materials and Methods section. Expression of CGA, CD56 (NCAM), SYN (p38), SSTR, and RB was detected by immunohistochemical staining.

^+^Secretion of CEA, CA19-9, and CA125 was tested in culture fluids by CLEIA at SRL Laboratories (Tokyo, Japan). The supernatant was collected from 48-h cultures.

TCC-NECT-2 cells were positive for neuroendocrine markers such as chromogranin A (CGA), cluster of differentiation 56 (neural cell adhesion molecule; CD56/NCAM), synaptophysin (major synaptic vesicle protein p38; SYN/p38), and neuron specific enolase (NSE), but not somatostatin receptor (SSTR). Expression of retinoblastoma (RB) protein was not detected in TCC-NECT-2 cells (Table 1). At the ultrastructural level, TCC-NECT-2 cells showed electron-dense, cytoplasmic, large dense-core neurosecretory granules that are typical of neuroendocrine cells as shown in Figure 1B.

TCC-NECT-2 cells secreted the tumor marker carbohydrate antigen (CA19-9) (41.6 ± 13.8 units/mL), but carcinoembryonic antigen (CEA) and carbohydrate antigen (CA125) production was not detected. These biological characteristics are summarized in Table 1. TCC-NECT-2 cells secreted large amounts (7510 pg/mL) of interleukin-8 (IL-8) and small amounts (2–4 pg/mL) of IL-4. Production of the following cytokines was not observed in these cells: IL-1β, IL-2, IL-3, IL-6, IL-10, vascular endothelial growth factor (VEGF), hepatocyte growth factor (HGF), and TP53 (data not shown).

Based on short tandem repeat (STR) genotyping, DNA extracted from the TCC-NECT-2 cell line did not correspond to cells in the database of the Japanese Collection of Research Bioresources (JCRB; a database of 2279 cells registered in the ATCC, the Deutsche Sammlung von Mikroorganismen und Zellkulturen, and the Japanese Collection of Research Bioresources).

### TCC-NECT-2-associated tumorigenicity and metastasis following different routes of implantation

Tumor formation was noted in nu/nu mice after the implantation of TCC-NECT-2 cells via subcutaneous (s.c.), intraperitoneal (i.p.), intra-duodenal, and intra-rectum routes with incidences of 88.9%, 83.3%, 83.3%, and 60%, respectively (Table 2). The mean survival period was 45.8 days for s.c., 47.7 days for i.p., 48.2 days for orthotopic, and 58.8 days for rectal implantation.

**Table 2:** Tumorigenicity and metastasis following different routes of implantation using the TCC-NECT-2 cell line

Cell line	Implantation route	Tumor formation^*^			Metastasis					Cachectic BW-loss (%)
		Frequency	Survival day	Histological pattern of xenografts^#^	Pancreas	Lymphnodes	Liver^⁑^	Stomach^$^	Peritoneal dissemination^+^	
TCC-NECT-2	s.c.	8/9	45.8±6.4	PD-NEC	0/8	0/8	0/8	0/8	0/8	2/8 (25)
	i.p.	5/6	47.7±8.3	PD-NEC	0/5	0/5	1/5	0/5	1/5	1/5 (20)
	Duodenum	5/6	48.2±4.8	PD-NEC	4/5	1/5	1/5	5/5	0/5	2/5 (40)
	Rectum	3/5	58.8±9.6	PD-NEC	0/3	0/3	0/3	0/3	0/3	1/3 (33)

^*^The tumorigenicity and metastasis of the cell lines were tested by subcutaneous (s.c.) injection of 1 × 10^6^ cultured cells suspended in 100 μL of PBS into mice. For the intraperitoneal (i.p.) inoculation of tumor cells, 2 × 10^6^ cells in 500 μL of PBS were injected into the abdominal cavity of the mice. For intra-duodenum or intra-rectum implantation of tumor cells, aliquots of 1 × 10^6^ cells in 50 μL of PBS were injected into the target organ of the mice. The mice were sacrificed on day 75 after tumor cell implantation or when they became moribund, and body weight (BW) loss was evaluated. Incidence is reported as fractions; numerators of each fraction indicate positive numbers in the samples (denominators).

^#^PD-NEC: poorly differentiated neuroendocrine carcinoma.

^⁑^Micrometastasis.

^$^Invasion from the duodenal tumor.

^+^Peritoneal dissemination: peritoneum, mesenterium, diaphragm.

The histological growth pattern of xenografts was as poorly differentiated NEC (polymorphic medullary type; Figure 1C and Table 2). TCC-NECT-2 cells exhibited strongly positive signals for CGA, CD56/NCAM, SYN/p38 (Figure 1D–1F), and Ki-67 (Table 1) based on immunohistochemical staining analysis.

**Figure 2:**
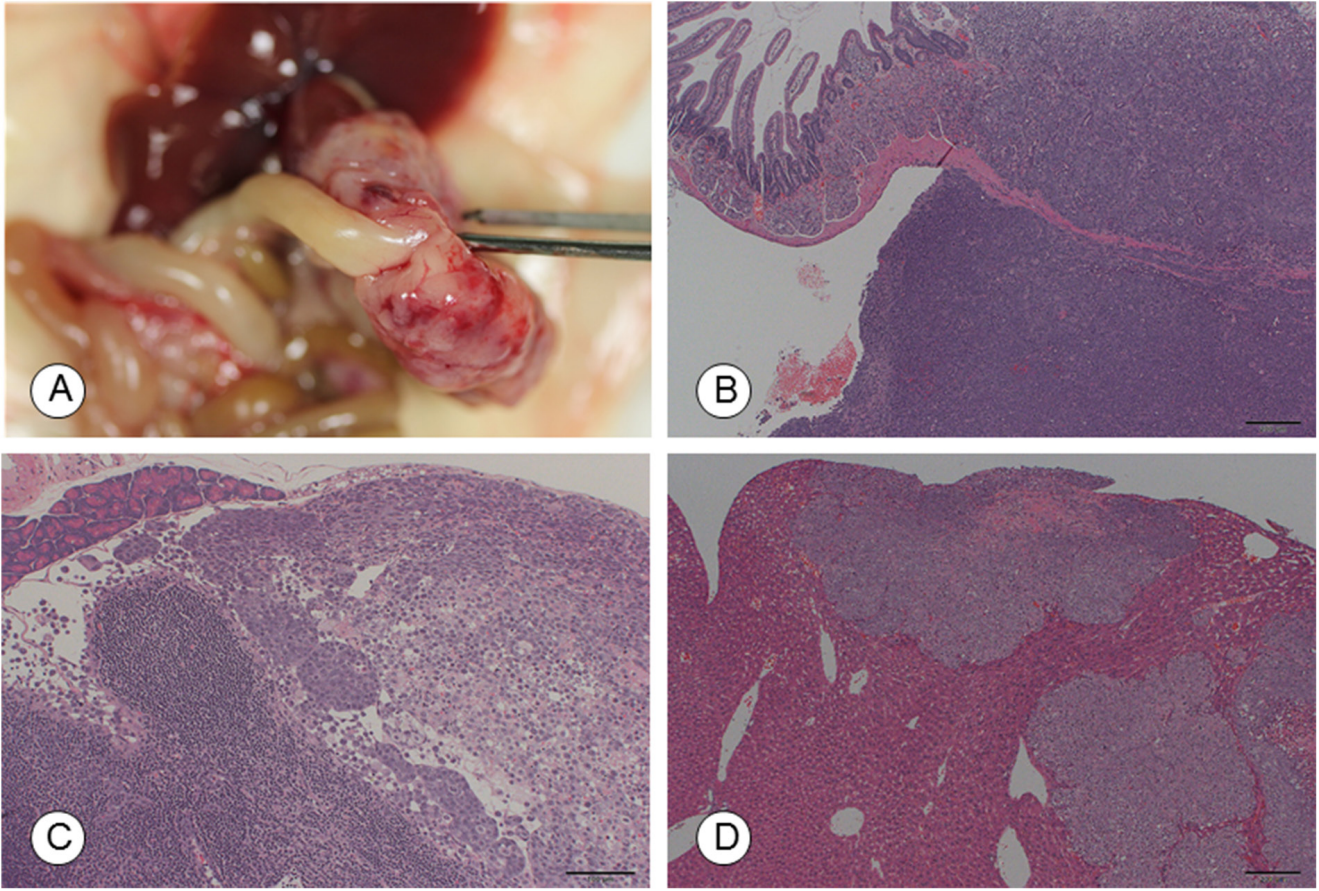
Macroscopic and microscopic photographs of tumors in the recipient mice following orthotopic implantation of TCC-NECT-2 cells **(A)** Photographs and **(B)** micrographs of the intra-duodenal tumor at 50 days post-orthotopic implantation. Scale bar: 200 μm, HE staining. **(C)** Lymph node metastasis. Scale bar: 100 μm. HE staining. **(D)** Micrometastasis in the liver at 50 days post-orthotopic implantation. Scale bar: 200 μm, HE staining.

Metastasis to the pancreas, lymph nodes, and liver, as well as invasion into the stomach, were noted only following orthotopic implantation at incidences of 20–100%. An intra-duodenal tumor mass and its histology, as well as lymph node metastasis, is shown in Figure 2A–2C. Liver micrometastasis was detected with orthotopic and i.p. implantation (Table 2 and Figure 2D). Peritoneal dissemination was observed after injection via the i.p. route at an incidence of 20%.

Interestingly, body weight reduction was observed in mice bearing TCC-NECT-2 xenografts at incidences of 25–40% (Table 2). The cachectic phenotype accompanied this body weight loss, including decreased activity, reductions in adipose tissue and musculature volumes, and decreases in the mass of other organs including the spleen and liver (data not shown).

### Molecular biological characterization of the TCC-NECT-2 cell line

Transcripts encoding gut hormones such as gastrin, insulin, glucagon, serotonin, and somatostatin were not expressed in TCC-NECT-2 cells based on real-time PCR analysis. Further, gut peptides such as gastric inhibitory polypeptide (GIP), vasoactive intestinal polypeptide (VIP), and motilin were undetectable at the mRNA level in TCC-NECT-2 cells (Supplementary Table 1 and Supplementary Figure 1).

Next, we performed next-generation sequencing analyses using the NCC oncopanel (v4) of 114 cancer-related genes (Supplementary Table 2) [14]. Mutations in BRAF^V600E^ and TP53 (Splicing783-1G>A) genes, as well as amplification of C-MYC (51-fold), were detected in TCC-NECT-2 cells. Further, the TP53 gene harbored the splicing mutation in both alleles. A KRAS gene mutation was not observed in this cell line.

### Inhibitory effect of vemurafenib and dabrafenib on the proliferation of BRAF^V600E^-expressing TCC-NECT-2 cells

**Figure 3:**
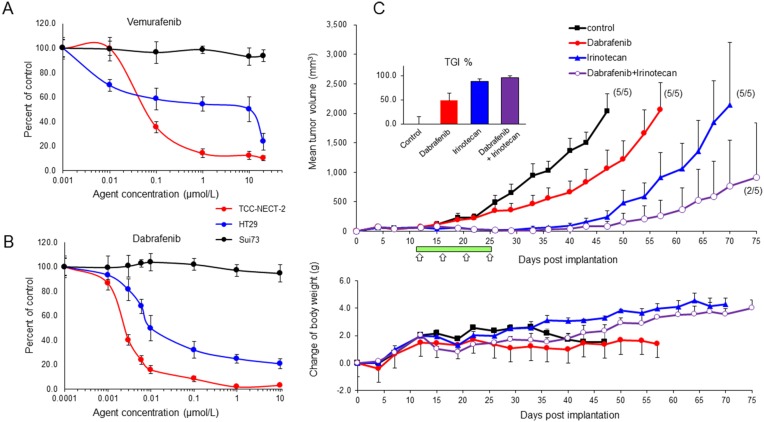
Effect of BRAF inhibitor on BRAF^V600E^-expressing TCC-NECT-2 cell proliferation and xenograft tumor growth Inhibitory effect of vemurafenib **(A)** and dabrafenib **(B)** on proliferation of TCC-NECT-2, HT29, and Sui73 cell lines; 2 × 10^4^ cells were seeded per well in 6-well plates, and exposed to vemurafenib or dabrafenib for 5 days. Each plotted value is the average ± SD. **(C)** Inhibition of TCC-NECT-2 xenograft tumor growth by dabrafenib or combination with irinotecan in nu/nu mice. Agents were administered orally for 14 days (green bar) starting on day 11 post-TCC-NECT-2 cell implantation as follows: dabrafenib at 30 mg/kg, once daily; irinotecan at 40 mg/kg, i.p. twice per week (white arrows). Tumor volumes and weights were recorded 2 to 3 times/week. Each plotted value is the average ± SEM for tumor volume and body weight. Numbers in parentheses: number of tumor-bearing mice/number of total mice; *n* = 5. The anti-tumor activity was assessed as the percent tumor growth inhibition (TGI%) in treated versus control mice (inserts). TGI represents the percent volume differential between treated and control tumors at the time when vehicle-treated tumors exceeded a volume of 2000 mm^3^ as described in the Materials and Methods section. TGI% is the average ± SEM.

The effect of two BRAF inhibitors, namely vemurafenib and dabrafenib, on cell proliferation was evaluated using the established BRAF^V600E^-expressing TCC-NECT-2 cell line and two human cancer cell lines including BRAF^V600E^-harboring HT29 colorectal cancer cells and BRAF^WT^-expressing Sui73 pancreatic cancer cells, which harbor a KRAS mutation (183A>C). Vemurafenib inhibited cell proliferation in a dose-dependent manner with IC_50_ values of 0.591 and 10.104 μmol/L for TCC-NECT-2 and HT29 cell lines, respectively (Figure 3A). However, it did not inhibit the proliferation of Sui73 cells, with an IC_50_ value of greater than 20.00 μmol/L. This observation is consistent with previous reports indicating that other cancer cell lines harboring BRAF^WT^, such as melanoma, colorectal carcinoma, or thyroid carcinoma cells, are insensitive to vemurafenib [15–17].

Next, we tested the growth suppressive effects of dabrafenib on BRAF^V600E^- and BRAF^WT^-expressing cancer cell lines. The inhibitory activity of dabrafenib was approximately 100-fold greater than that of vemurafenib with BRAF^V600E^-harboring cancer cell lines. Dabrafenib strongly suppressed cell proliferation with IC_50_ values 0.0025 and 0.0121 μmol/L for TCC-NECT-2 and HT29 cell lines, respectively. In addition, the Sui73 cell line was insensitive to dabrafenib (IC_50_ value greater than 10.00 μmol/L). These data are shown in Figure 3B.

### Efficacy of dabrafenib mono- and combination-therapy in the BRAF^V600E^-expressing TCC-NECT-2 xenograft model

Based on the *in vitro* results, we tested the anti-tumor activity of dabrafenib using animal experiments. We explored combination therapies, with the standard therapeutic irinotecan, because selective inhibitors have shown limited single-agent clinical activity in BRAF^V600E^-mutant metastatic melanoma [18, 19].

The *in vivo* anti-tumor activity of dabrafenib alone or in combination with irinotecan was tested in a TCC-NECT-2 xenograft model. Mice were dosed orally once daily at 30 mg/kg of dabrafenib for 14 days or that combined with 40 mg/kg of irinotecan four times, and tumor volumes were measured until the endpoint (75 days) (Figure 3C). When tumor volumes reached 2000 mm^3^, as the limit of observed tumor growth, mice in each experimental group were sacrificed. Tumor growth inhibition was presented as the percent volume difference between treated and control tumors at the time when vehicle-treated tumors exceeded 2000 mm^3^. Figure 3C (top) shows the tumor growth curve (average of five animals). With treatment, the inhibition of tumor growth was significant when compared to vehicle-treated control tumor volumes at 47 days post-implantation; the percent tumor growth inhibition (TGI%) was 48.04, 87.97, and 95.81, with p-values of 0.0434, 0.0011, and 0.0006, for dabrafenib, irinotecan, and combination groups, respectively (Figure 3C, inserts).

In every case, the cessation of drug treatment resulted in tumor outgrowth; however, the time required to reach the tumor volume limit was markedly longer in the drug treated groups compared to that in the vehicle-treated control group. In the dabrafenib treatment group, tumor growth occurred slowly throughout treatment, but the time required to reach the maximum volume was longer compared to that in the control group. In contrast, in the combination and irinotecan-treated groups, tumor growth was strongly suppressed until day 43, with no sign of tumor growth at that time. However, tumor growth resumed at approximately day 50, and tumor volume reached the maximum value in the irinotecan treatment group on day 69. Three of five mice showed complete tumor regression in the combination group at day 75 (endpoint of this experiment). Thus, with these drugs, TCC-NECT-2 tumor growth was strongly suppressed (Figure 3C).

Body weights in the irinotecan and dabrafenib/irinotecan treatment groups increased gradually until the endpoint of the study (Figure 3C lower). However, body weights in the dabrafenib treatment and vehicle-treated control groups did not differ noticeably throughout the study; the body weight at the beginning and endpoint of the trial was 18.4–22.3g and 24.8–25.5 g, respectively (average of five animals).

## DISCUSSION

Our study presents two major findings. First, we established and characterized a human NEC cell line from duodenal cancer. Second, we determined the anti-proliferation effect of vemurafenib and dabrafenib on BRAF^V600E^-expressing TCC-NECT-2 cells *in vitro*. In addition, we discovered that dabrafenib significantly suppresses tumor growth in a xenograft model, and has an enhanced therapeutic effect when combined with irinotecan.

NEC is a highly malignant rare carcinoma that occurs in various organs such as digestive and respiratory organs, and an increasing incidence for this disease has been reported in recent years [20]. In particular, since D-NEC is a very rare cancer (incidence rate of 0.06–2.9%) among digestive cancers [7, 8], reports of associated genetic alterations and the carcinogenic mechanisms are scarce. Research into new treatment options is active, but remains in the stage of review for standardization [9, 10]. A D-NEC derived cell line is thus indispensable for studies on carcinogenic mechanisms and molecular target discovery, but this cell line has not yet been described [13]. The TCC-NECT-2 cell line, derived from D-NEC, which we established in this study, will be a valuable asset as an experimental model for basic and preclinical research.

TCC-NECT-2 cells were positive for neuroendocrine markers (CGA, SYN/p38, CD56/NCAM, and NSE) and were confirmed to be derived from NEC. However, digestive tract hormones and peptides were not detected. Considering the tumorigenicity, metastasis, high Ki-67 index, and RB protein loss observed with TCC-NECT-2 cells, the established cell line comprised a poorly differentiated NEC [10, 21, 22] and was confirmed to be hormonally inactive [10, 23, 24].

As mentioned previously, the importance and usefulness of OI models in translational research is well known [25, 26]. In fact, our previous reports of OI with gastric carcinoma cells in mice showed that subsequent tumor growth results in metastases to various organs and cachexia syndrome, as observed in human cases [11, 12]. We attempted TCC-NECT-2 cell implantation using s.c., i.p., rectal, and duodenal routes and compared the tumorigenesis and metastasis/invasiveness in this study. Tumorigenicity and metastasis were frequently observed with OI, as compared to the rates observed with other routes, and thus the usefulness of this approach was confirmed, as this method effectively mimicked the tumor/host interaction and pathogenesis of aggressive D-NEC [8, 23, 25].

Recent clinical sequencing data has improved our understanding of NETs and has provided critical information for the development of new therapeutic agents in this field. However, the precise genetic background of poorly differentiated NEC is still unknown [27]. Only a few reports have described genetic alterations based on immunohistochemical and target-sequencing analysis of GEP-NEC, and most of these studies were performed on NEC from a specific organ such as the pancreas [27–29] or colon [30, 31]. Predominantly identified gene mutations were as follows: TP53, RB1, KRAS, CDKN2A, CDKN2B, MEN1, DAXX, APC, and BRAF. We discovered a TP53 splicing mutation in both alleles of the established TCC-NECT-2 cell line. TP53 is mutated in many types of cancer, and various mutations have been identified during tumorigenesis and metastasis. Interestingly, we also identified a BRAF^V600E^ mutation in this cell line. BRAF^V600E^ is an established oncogenic driver and is mutated in a variety of human cancers including malignant melanomas (mutation rate of 50%), thyroid cancers (40%), colon cancers (12%), and ovarian cancers (7%) [32–34]. In contrast, few studies have performed mutational analyses of BRAF in gastrointestinal NECs to date. A recent report identified a 20% BRAF mutation rate (two V600E) and 40% KRAS mutation rate (two G12D, one G12V, and one G13D) in patients with colorectal NEC based on a retrospective series of 10 patients [35]. Klempner et al. reported a 9% rate of BRAF mutations in 108 cases of high-grade colorectal NECs, of which 80% were V600E mutations [36]. More recently, Idrees et al reported a 44% BRAF mutation rate in nine cases of colonic NEC, three of which were V600E mutations, and one of which was a D594G mutation [37].

BRAF, a serine/threonine kinase that is commonly activated by somatic point mutations in human cancer, could provide new therapeutic opportunities for NEC. Currently, BRAF/MEK inhibitor combination therapy is FDA-approved for the treatment of melanoma. Klempner et al reported a dramatic response to BRAF/MEK inhibitor combination therapy, which occurred in two cases of metastatic colorectal NEC harboring BRAF^V600E^ substitutions that were refractory to standard therapy [36]. This paper suggested that BRAF^V600E^ might provide new therapeutic opportunities as a druggable oncogene in D-NEC.

The selective BRAF inhibitor vemurafenib has produced a dramatic response rate (> 50%) in BRAF-mutant metastatic melanomas, and is currently used as the standard of care for this disease [38]. However, single-drug treatment frequently results in acquired resistance after a median response duration of 6–7 months [39]. Dabrafenib is a selective, potent, ATP-competitive inhibitor of the BRAF^V600E^-mutant kinase that has demonstrated efficacy in clinical trials. TCC-NECT-2 cells were sensitive to vemurafenib and dabrafenib *in vitro*, and were especially sensitive to dabrafenib. Using a TCC-NECT-2 xenograft model, we therefore explored dabrafenib combination therapy, with the standard therapeutic agent irinotecan, because it has shown limited single-agent clinical activity in BRAF^V600E^-mutant metastatic colorectal cancer [40, 41]. As a result, the addition of irinotecan to dabrafenib resulted in increased anti-tumor activity in this model. It is suggested that BRAF^V600E^ mutant D-NEC, for which there is currently no targeted treatment options available, might benefit from combination therapy comprising a BRAF inhibitor and standard chemotherapy agent. One limitation of this study was that it was performed using only one established cell line. To overcome this limitation and confirm our findings, we are attempting to establish more cell lines and patient-derived xenografts.

Additionally, in TCC-NECT-2 tumor-bearing mice, body weight loss, decreased activity, skin dryness, and anorexia were observed with low frequency. Thus, the TCC-NECT-2 cell line might provide a useful model for studying the basic biology of cachexia [42, 43].

In conclusion, we established and characterized a human D-NEC cell line. To our knowledge, the TCC-NECT-2 cell line is the first cell line that has been derived from D-NEC. This *in vitro* and *in vivo* model represents a promising tool to analyze the pathobiology of this rare disease, which could facilitate the discovery of therapeutic targets and molecules.

## MATERIALS AND METHODS

### Origin and establishment of TCC-NECT-2 cell line

The patient, a 59-year-old Japanese man, was diagnosed with NEC of the duodenum through histological examination of tissue, which was composed of the argyrophil neoplastic cells immunohistochemically positive for the following distinct epithelial and neuroendocrine markers: grimelius, CGA, NSE, somatostatin, serotonin, keratin, and vimentin. The patient had received short-term chemotherapy (details unavailable).

The TCC-NECT-2 cell line was established according to our routine protocol of peritoneal effusion obtained by peritoneocentesis from a patient [44, 45]. Briefly, after the collection of ascitic tumor cells via centrifugation (760 × g for 10 min), tumor cells were seeded into 100-mm culture dishes (Falcon, New York, USA) containing DMEM (Dulbecco's Modified Eagle Medium) supplemented with 10% FBS (Gibco, California, USA) and 1% penicillin/streptomycin (Gibco). They were maintained at 37 °C in a humidified incubator with 5% CO_2_. The primary culture was first split after 3 months of cultivation, and thereafter the cells were passaged to 60–80% confluence at a ratio of 1:10. The culture was then judged, established, and designated (TCC-NECT-2) in 1998, and stored in liquid nitrogen. The cryopreserved cells were thawed routinely in 2010 and used in this study. The cell line was regularly tested for Mycoplasma using a PCR Mycoplasma Detection kit (Takara, Shiga, Japan), and no contamination was detected. This study was conducted in accordance with the Declaration of Helsinki. Informed consent was obtained from the patient. The study protocol was approved by the local ethics committees.

### Tumor markers and cytokines

Tumor cells (1 × 10^6^ cells) were seeded in 100-mm dishes with DMEM supplemented with 10% FBS and cultured for 2 days. The medium was then replaced. After 1 day, the culture supernatant (1.5 × 10^6^ cells/mL) was collected and centrifuged at 1710 × g for 10 min to eliminate cell debris. The resulting supernatant was stored at −80 °C until use in assays. Concentrations of CEA, CA19-9, CA125, and NSE were determined by the chemiluminescent enzyme immunoassay (CLEIA) at SRL Laboratories (Tokyo, Japan). Secretion of IL-1β, IL-2, IL-3, IL-8, IL-10, VEGF, HGF, and TP53 was tested by enzyme-linked immunosorbent assay (ELISA) at FALCO Biosystems (Kyoto, Japan). Secretion of IL-4 and IL-6 was tested by CLEIA. The results are mean values of triplicate assays (variability less than 10%).

### Short tandem repeat analysis

STR genotyping was performed using genomic DNA extracted from the TCC-NECT-2 cell line. This analysis was performed by Promega (Tokyo, Japan). This experiment was conducted using the PowerPlex® 16 System (Promega) according to the manufacturer's instructions. The cell authentication report number of the cell lines established in this study is KBN 0299.

### Animal experimentation

All procedures in this study involving animals and their care were approved by the Committee for Ethics in Animal Experimentation of Yasuda Women's University and the National Cancer Center in accordance with Institutional and Japanese Government Guidelines for Animal Experiments. Female BALB/c nu/nu mice were purchased from CLEA Japan (Tokyo, Japan) and maintained under specific pathogen-free conditions. Six to eight-week-old mice (18–22 grams) were used for these experiments.

For OI, after the induction of anesthesia with 5 % isoflurane in room air (flow, 300 mL/min), mice were maintained in 2% isoflurane anesthesia via a face mask throughout the operation. After sterilization of the abdomen with 70% ethanol, a small incision was made in the median abdominal wall under anesthesia and the duodenum was exposed; 1 × 10^6^ cells in 50 μL of PBS were directly injected into the duodenum of the mice using a 30-gauge needle (Nipro Co, Tokyo, Japan). For implantation into the rectum, tumor cells (1 × 10^6^ cells in 50 μL of PBS) were inoculated into the middle wall of the rectum using a 30-gauge needle. The needle was carefully withdrawn to avoid regurgitation along the needle track and the injection orifice was pressure-sealed with a dry cotton tip. The incised abdominal wall was closed with an AUTOCLIP Applier (Becton Dickinson, Maryland, USA). After confirming recovery from bradycardia and stable spontaneous respiration, the mice were returned to their cages. The mice were sacrificed when tumor volumes reached 2000 mm^3^ (the limit of tumor growth), or when animals became moribund. Abdominal tissues were inspected macroscopically for metastasis examining various organs and thereafter processed for histological examination, as described previously [44].

### Pathomorphological and immunohistochemical analyses

Tumor tissues from mice transplanted with cancer cells were fixed in phosphate-buffered 10% formalin and embedded in paraffin. Sections were cut at 5-μm intervals and stained with hematoxylin-eosin according to routine histological protocols. Ultrastructural studies were performed on the cells as previously reported [46]. Immunohistochemical staining was performed according to the manufacturer's instructions and/or standard protocols, as described previously [47]. Antibodies used included: anti-CGA (1:500) from Neomarker (California, USA); anti-Ki-67 (1:250), anti-serotonin (1:200), anti-cytokeratin (AE1/AE3 1:50), anti-vimentin (M0725 1:100), and anti-synaptophysin (1:200), all from Dako (California, USA); anti-somatostatin receptor 2A (1:500) and 5 (1:500) from Gramsch (Schwabhausen, Germany); anti-CD56/NCAM (1:100) from Novocastra (Newcastle, England); anti-RB (clone 3H9, 1:300) was from MBL (Nagoya, Japan). VECTASTAIN ABC HRP kit, from Vector Laboratories (California, USA), was used for the analysis. The Ki-67 index was obtained by counting the ratio of Ki-67 positive cells versus total nuclei using VENTANA iScan HT (Arizona, USA).

### Next generation sequencing

Genomic DNA extracted from the TCC-NECT-2 cell line was prepared using a QIAamp DNA Mini Kit (Qiagen, Hilden, Germany) according to the manufacturer's protocol. We performed next generation sequence analyses using the NCC oncopanel (v4) for 114 cancer-related genes (listed in Supplementary Table 1). Targeted sequencing and data analysis were previously described [14].

### Cell lines, regents, and cellular proliferation assays

The Sui73 cell line was established in our laboratory [45]. The HT29 cell line was purchased from the American Type Culture Collection. All cell lines were passaged for fewer than 3 months from the stocks of first or second passages of the original clones and were authenticated by sequencing to determine the status of BRAF. All cell lines were maintained in DMEM supplemented with 10% heat-inactivated FBS (Gibco). Vemurafenib (Cayman, Michigan, USA) was prepared at a 10× stock relative to the final assay concentration in media containing 0.5% dimethyl sulfoxide (DMSO) [17]. Dabrafenib (Selleckchem, Texas, USA) was formulated as a suspension with 0.1% DMSO in PBS (Gibco) [47]. To analyze the sensitivity to each agent, cells were seeded at a density of 2 × 10^4^ cells/well in flat bottom 6-well plates (Falcon). On the next day, a dilution series containing the appropriate drug concentrations was applied and cells were incubated for 5 days in a humidified incubator with an atmosphere of 5% CO_2_ at 37 °C. Cell proliferation was estimated using the TC20 automated cell counter (Bio-Rad, Tokyo, Japan). The cell number was counted, and results were expressed as the mean percentage of triplicate measurements. All experiments were performed independently and repeated three times. Percent relative to the control was calculated using the formula: percent of control = (each cell number in experimental wells/mean cell number of control wells) × 100. The IC_50_ was determined based on the regression of a plot of the logarithmic concentration versus percent inhibition using JMP (version 11.2; SAS, NC, USA) and a dose–response one-site model.

### Efficiency of dabrafenib mono- and combination-therapy in a BRAF^V600E^-expressing TCC-NECT-2 xenograft model

TCC-NECT-2 cells (5 × 10^6^ cells/100 μL) were implanted subcutaneously in female BALB/c nu/nu mice and grown to form tumors. When tumors reached 150–200 mm^3^, five animals were randomly assigned to each treatment group as follows: (1) vehicle; 0.5% hydroxylpropyl-methylcellulose and 0.2% tween 80 in pH 8.0 distilled water; (2) dabrafenib; 30 mg/kg, oral-gavage/once daily, twice per week; (3) irinotecan; 40 mg/kg, via i.p, twice per week; (4) combination dabrafenib and irinotecan. Irinotecan/CPT-11 (Towa, Osaka, Japan) was provided as a sterile stock solution in saline of 20 mg/mL, which was diluted as required with sterile saline. Treatment was continued for 2 weeks, and tumor growth was evaluated twice weekly by measuring tumor diameters with a two-dimensional caliper; tumor volume (TV) was calculated according to the following formula: v = (l × w^2^/2), where v = volume (mm^3^), l = length (mm), and w = width (mm); this was reported as the mean value of five mice per group. The anti-tumor activity was assessed as TGI% in treated versus control mice, calculated as follows: TGI % = 100 − (TV of each treated tumor/mean TV of vehicle control group × 100). TGI represents the percent volume differential between treated and control tumors at the time when vehicle-treated tumors exceeded a volume of 2000 mm^3^. Mice were sacrificed if they had a tumor greater than 1.6 cm in diameter (exceeding a volume of 2000 mm^3^), if total tumor burden was greater than 10% of body weight, or if a tumor became ulcerated or interfered with mobility. All mice were sacrificed 75 days post-tumor cell inoculation as endpoint of the experiment. The experiments were performed in accordance with the Institutional and Japanese Government Guidelines for Animal Experiments.

### Statistical analysis

All data were analyzed using the unpaired t-test and expressed as mean ± SD for *in vitro* assays and as mean ± SEM for *in vivo* analysis; p-values less than 0.05 were considered statistically significant.

## SUPPLEMENTARY MATERIALS FIGURE AND TABLES



## Abbreviations

D-NEC: neuroendocrine carcinoma of the duodenum
CGA: chromogranin A
CD56: cluster of differentiation 56
SYN: synaptophysin
NSE: neuron specific enolase
NET: neuroendocrine tumor
RB: retinoblastoma
GEP: gastroenteropancreatic
OI: orthotopic implantation
SSTR: somatostatin receptor
STR: short tandem repeat
HGF: hepatocyte growth factor
IL: interleukin
VEGF: vascular endothelial growth factor
s.c.: subcutaneous
i.p.: intraperitoneal
GIP: gastric inhibitory polypeptide
VIP: vasoactive intestinal polypeptide
TGI: tumor growth inhibition
DMEM: Dulbecco's modified eagle medium
CEA: carcinoembryonic antigen
CLEIA: chemiluminescent enzyme immunoassay
ELISA: enzyme-linked immunosorbent assay
DMSO: dimethyl sulfoxide
PBS: phosphate buffered saline
TV: tumor volume
PD-NEC: poorly differentiated neuroendocrine carcinoma
