# Supplementary material for: Establishment of a novel cell line from a rare human duodenal poorly differentiated neuroendocrine carcinoma
Source: Oncotarget. 2018 Nov 23;9(92):36503–14. doi: 10.18632/oncotarget.26367 (PMC6284856; doi:10.18632/oncotarget.26367)
Supplement: Supplementary file 1 [file oncotarget-09-36503-s001.pdf]

Establishment of a novel cell line from a rare human duodenal poorly differentiated neuroendocrine carcinoma

SUPPLEMENTARY MATERIALS

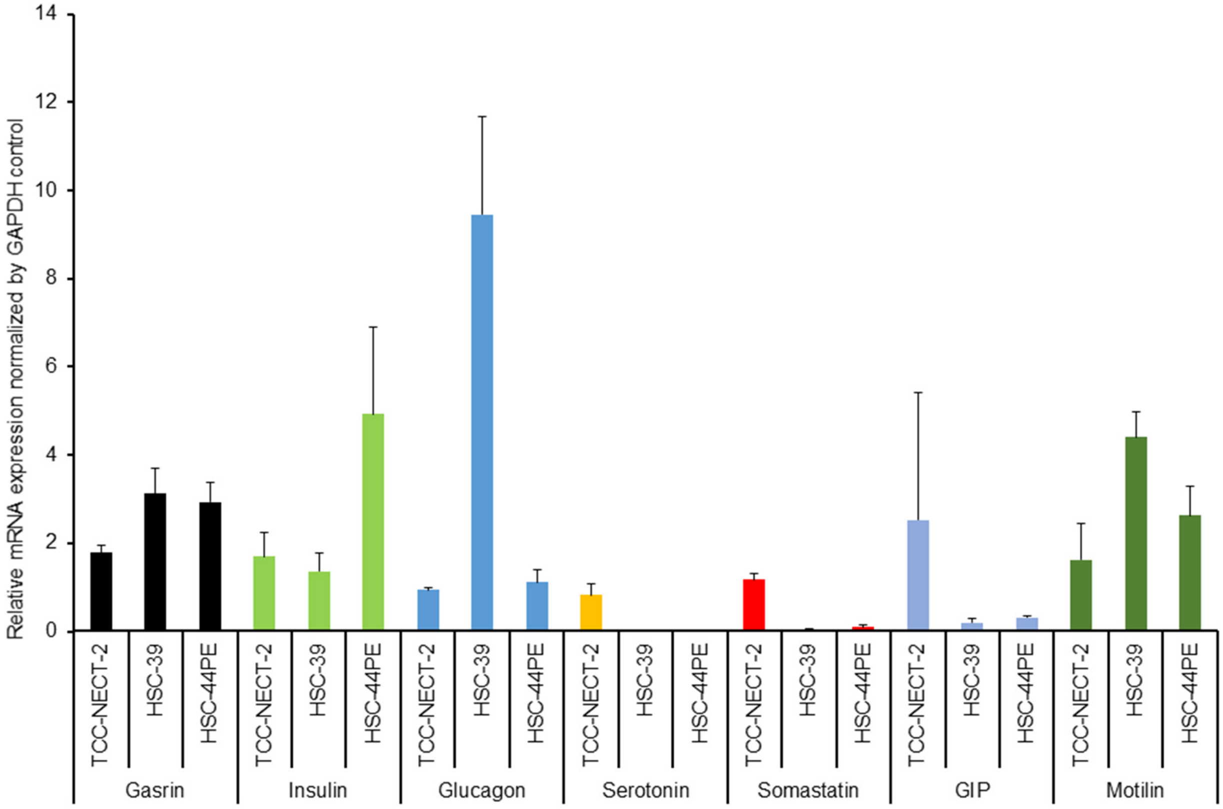

Supplementary Figure 1: Gene expression levels of various gut-hormones and -peptides. HSC-39 and HSC-44PE human gastric cell lines were employed as controls. Relative mRNA expression level of serotonin was 0.0004 for HSC-39 and HSC-44PE cells, respectively [1]. Expression of VIP was not detected in all cell lines (data not shown).

Supplementary Table 1: Target genes and sequence of primers used for real-time PCR (RT-PCR)

Genes	Forward (5′ → 3′)	Reverse (5′ → 3′)
Gastrin	ATCTGCCTGACATCCACG	CCAGCTGCCTTCGATGA
Insulin	CCAGCCGCAGCCTTTGTGAA	AGCAATGGGCGGTTGGCTCA
Glucagon	GAATTCATTGCTTGGCTGGT	GGCGGCAAGATTATCAAGAA
Serotonin	CCTCTCACCGTCGTGTCTCT	GTGGCATTCTGCAGCTTTTT
Somatostatin	AACCAGACGGAGAATGATGC	CCATAGCCGGGTTTGAGTTA
GIP	GAAATCAGGGGAAGGAGACC	GCCACCATCTTCCAAGGTTA
VIP	TGGGCGAGGTAAGTGAAAAC	CATGCCGAAGTCTGTGAGAA
Motilin	GTTTTCAGTGGACGGGAGAA	GAAGCTCGTGGAAACTCCAG
GAPDH	ACGACCAAATCCGTTGACTC	GCTCTCTGCTCCTCCTGTTC

The level of gene expression was evaluated by measuring target mRNAs quantitatively using reverse transcription and real-time PCR. Glyceraldehyde-3-phosphate dehydrogenase (GAPDH) mRNA was also measured as an intrinsic control. The total RNA was extracted using an RNeasy Plus Mini Kit (Qiagen, Valencia, CA), and the yield was determined by measuring the optical density at a wavelength of 260 nm on a UV spectrometer. Complementary DNA was synthesized using the PrimeScript™ RT reagent Kit (Takara, Shiga, Japan) using 20 μg/mL of extracted total RNA. Real-time PCR was performed using a Thermal Cycler Dice Real Time System II (Takara) with the SYBR Premix EX Taq™ II (Takara) reagent according to the manufacturer's protocol.

Supplementary Table 2: Gene list of NCC-oncopanel ver. 4

Gene symbol				
SNV, Amplification (114 genes)				Gene fusion (12 genes)
ABL1	EGFR	KRAS	PIK3CA	ALK
ACTN4	ENO1	MAP2K1/MEK1	PIK3R1	AKT2
AKT1	EP300	MAP2K2/MEK2	PIK3R2	BRAF
AKT2	ERBB2/HER2	MAP2K4	POLD1	ERBB4
AKT3	ERBB3	MAP3K1	POLE	FGFR2
ALK	ERBB4	MAP3K4	PRKCI	FGFR3
APC	ESR1/ER	MDM2	PTCH1	NRG1
ARAF	EZH2	MDM4	PTEN	NTRK1
ARID1A	FBXW7	MET	RAC1	NTRK2
ARID2	FGFR1	MLH1	RAC2	PDGFRA
ATM	FGFR2	MTOR	RAD51C	RET
AXIN1	FGFR3	MSH2	RAF1/CRAF	ROS1
AXL	FGFR4	MYC	RB1	
BAP1	FLT3	MYCN	RET	
BARD1	GNA11	NF1	RHOA	
BCL2L11/BIM	GNAQ	NFE2L2/Nrf2	ROS1	
BRAF	GNAS	NOTCH1	SETBP1	
BRCA1	HRAS	NOTCH2	SETD2	
BRCA2	IDH1	NOTCH3	SMAD4	
CCND1	IDH2	NRAS	SMARCA4/BRG1	
CD274/PD-L1	IGF1R	NRG1	SMARCB1	
CDK4	IGF2	NTRK1	SMO	
CDKN2A	IL7R	NTRK2	STAT3	
CHEK2	JAK1	NTRK3	STK11/LKB1	
CRKL	JAK2	NT5C2	TP53	
CREBBP	JAK3	PALB2	TSC1	
CTNNB1/b-catenin	KDM6A/UTX	PBRM1	VHL	
CUL3	KEAP1	PDGFRA		
DDR2	KIT	PDGFRB		

We performed next generation sequence analyses using the NCC-oncopanel (v4) for 114 cancer-related genes. Targeted sequencing and data analysis were previously described [2].

REFERENCES

1. Yanagihara K, Tanaka H, Takigahira M, Ino Y, Yamaguchi Y, Toge T, Sugano K, Hirohashi S. Establishment of two cell lines from human gastric scirrhous carcinoma that possess the potential to metastasize spontaneously in nude mice. Cancer Sci. 2004; 95:575–82. https://doi.org/10.1111/j.1349-7006.2004.tb02489.x.
2. Tanabe Y, Ichikawa H, Kohno T, Yoshida H, Kubo T, Kato M, Iwasa S, Ochiai A, Yamamoto N, Fujiwara Y, Tamura K. Comprehensive screening of target molecules by next-generation sequencing in patients with malignant solid tumors: guiding entry into phase I clinical trials. Mol Cancer. 2016; 15:73. https://doi.org/10.1186/s12943-016-0553-z.
